# Association of sonic hedgehog signaling pathway genes IHH, BOC, RAB23a and MIR195-5p, MIR509-3-5p, MIR6738-3p with gastric cancer stage

**DOI:** 10.1038/s41598-021-86946-0

**Published:** 2021-04-02

**Authors:** Sadegh Fattahi, Novin Nikbakhsh, Mohammad Ranaei, Davood Sabour, Haleh Akhavan-Niaki

**Affiliations:** 1 Cellular and Molecular Biology Research Center, Health Research Institute, Babol University of Medical Sciences, Babol, Iran; 2 Department of Surgery, Rouhani Hospital Babol University of Medical Sciences, Babol, Iran; 3 Department of Pathology, Rouhani Hospital, Babol University of Medical Sciences, Babol, Iran; 4 Department of Genetics, Faculty of Medicine, Babol University of Medical Sciences, Babol, Iran

**Keywords:** Gastric cancer, Biomarkers

## Abstract

Gastric cancer is the leading cause of cancer-related mortality worldwide. Given the importance of gastric cancer in public health, identifying biomarkers associated with disease onset is an important part of precision medicine. The hedgehog signaling pathway is considered as one of the most significant widespread pathways of intracellular signaling in the early events of embryonic development. This pathway contributes also to the maintenance of pluripotency of cancer stem cells pluripotency. In this study, we analyzed the expression levels of sonic hedgehog (Shh) signaling pathway genes IHH, BOC, RAB23a and their regulatory miRNAs including MIR-195-5p, MIR-509-3-5p, MIR-6738-3p in gastric cancer patients. In addition, the impact of infection status on the expression level of those genes and their regulatory miRNAs was investigated. One hundred samples taken from 50 gastric cancer patients (50 tumoral tissues and their adjacent non-tumoral counterparts) were included in this study. There was a significant difference in all studied genes and miRNAs in tumoral tissues in comparison with their adjacent non-tumoral counterparts. The lower expression of IHH, BOC, RAB23, miR-195-5p, and miR-6738-3p was significantly associated with more advanced cancer stage. Additionally, IHH upregulation was significantly associated with CMV infection (*P* < 0.001). Also, receiver operating characteristic (ROC) curve analysis indicated that mir-195 was significantly related to several clinicopathological features including tumor stage, grade, age, gender, and infection status of gastric cancer and can be considered as a potential diagnostic biomarker for gastric cancer. This study confirms the important role of Shh signaling pathway genes in gastric cancer tumorigenesis and their potential as novel molecular biomarkers and therapeutic targets.

## Introduction

Gastric cancer is among the most frequently diagnosed neoplasia in both sexes and the third leading cause of death worldwide, causing about 1,000,000 new cases and 783,000 deaths in 2018^1^. Remarkably, it has been shown that gastric cancer, similar to other types of cancer, develops upon accumulation of genetic and epigenetic abnormalities^2–4^. These alterations can be triggered by a wide variety of external factors such as diet, life style, and infections^5–8^. Relatively, genes that play important roles in various cellular processes via cell signaling pathways could be affected^9,10^. Inappropriate activation of one or several signaling pathways may contribute to tumor initiation and progression^11,12^. The sonic hedgehog (Shh) is considered as one of the most significant intracellular signaling cascades in the early events of embryonic development that regulate a wide range of biological functions, such as cell growth, differentiation, tissue patterning, and vascularisation^13,14^. Moreover, emerging evidence has suggested that aberrant activation of hedgehog (*Hh*) is associated with a variety of human cancers such as brain, gastrointestinal, lung, breast and prostate cancers as well as the maintenance of cancer stem cells^15–20^. Two Shh signaling pathways have been characterized: a canonical pathway that is ligand-dependent, and a non-canonical ligand-independent pathway. In the canonical Shh signaling pathway, the Hh proteins, sonic (SHH), Indian (IHH), and desert (DHH) hedgehogs bind to the receptor patched (PTCH1), which inhibits SMO, allowing SMO accumulation and translocation of activated forms of GLI family zinc finger 1 (GLI) into the nucleus, where it mediates the transcription of Hh target genes^21^. Furthermore, Hh co-receptors cell-adhesion-molecule-related CDON) and biregional CDON -binding protein (BOC), which encode cell surface bound members of the Ig/fibronectin-domain-superfamily, can also promote Shh signaling^22,23^. Also, Shh signaling can control through pathway negative regulators including Hh-interacting protein1 (HHIP) and RAB23, member RAS oncogene family (RAB23)^24–26^. In addition, post-transcriptional and/or translational regulations of Shh genes through micro RNAs (miRNAs) have been reported^27,28^. Furthermore, viral infections were shown to play an important role in Shh signaling pathway activation^29–31^. In a previous study, we unveiled important signaling pathways involved in the progression of gastric cancer using RNA-seq technology. The aim of this study was to evaluate the expression levels of genes associated with Shh signaling pathway that were significantly modulated and showed at least fourfold expression change, using real time PCR in gastric cancerous and non-cancerous tissues. In addition, for those dysregulated genes, we further evaluated their associations with expression levels of regulatory miRNAs and infection status.

## Materials and methods

### Patients

Tissue specimens used in the present study, including 50 tumoral and their adjacent non-tumoral tissues were collected during September 2015 to June 2018 at Mazandaran province, Iran hospitals. All samples were cut into approximately 0.5 cm^3^ pieces and individually snap-frozen in liquid nitrogen immediately after surgery, and thereafter stored at − 80 °C without thawing for further analyzes. Diagnosis of all cases was histologically confirmed by a pathologist using hematoxylin and eosin (H&E) staining, and infections including *H. pylori*, cytomegalovirus (CMV), human herpesvirus 6 (HHV6), and Epstein-Barr virus (EBV) were evaluated by PCR and Real-time PCR, as previously described^32^. Informed consent was obtained from all patients, and all procedures were approved and carried out in accordance with the guidelines of the Ethics Committee of Babol University of Medical Sciences, and were performed in compliance with Helsinki declaration.

### RNA extraction

Tumoral and their adjacent non-tumoral tissues were separately crushed and homogenized with liquid nitrogen. RNX-plus reagent (Cinagen, Iran) was used to extract total RNA from samples according to the standard protocol with small modifications. Briefly, tumors were homogenized using 1 ml of RNX-plus per 10 mg of tissue. 200 μl of chloroform were added per 1 mL of RNX-plus for each sample. Samples were incubated for 10 min at room temperature and centrifuged for 15 min at 12,000×*g* at 4 °C. The aqueous phase containing the RNA was transferred into a new tube. Then 500 μl isopropanol was added, and the solution was incubated for 1 h and centrifuged for 40 min at 12,000×*g* at 4 °C. The supernatant was then discarded, and the pellet containing the RNA was washed with 500 μl 75% ethanol, left to dry for 10 min, and resuspended in 40 μl RNase-free water. The purified RNA samples were visualized after electrophoresis on 1.8% agarose gel to monitor the RNA integrity.

### Prediction of miRNAs targeting BOC, IHH, and RAB23

Twelve online sequence-based target prediction algorithms have been used to predict miRNAs targeting *BOC*, *IHH*, and *RAB23* mRNAs. MiRNAs that were predicted in at least seven out of twelve databases were selected for further analysis. MiR-6738-3p, miR-195-5p and miR-509-3-5p targeting the 3′′UTR of the *BOC*, *IHH*, and *RAB23* mRNAs, respectively.

### Quantitative real-time PCR analysis (QPCR)

Reverse transcription was performed using total RNA with gene specific stem-loop primer^33,34^ and SuperScript II reverse transcriptase (Invitrogen, USA). QRT-PCR was performed with the gene-specific primers listed in Table 1 using HotStar-Taq DNA Polymerase kit (Qiagen, Germany) in triplicate on the StepOne Plus real time instrument (Applied Biosystems, USA) according to the manufacturer’s instructions, to detect the expression levels of the target mRNAs and Micro-RNAs. The amplification conditions were 15 min at 95 °C, 40 cycles of 20 s at 95 °C followed by 60 s at 60 °C. The expression levels of mRNAs and miRNAs were normalized to Glyceraldehyde-3-phosphate dehydrogenase *(GAPDH* and RNA U6 small nuclear 1 (*RNU6)*, respectively. The sequence of forward and reverse primers along with universal Taqman probe is presented in Table 1. The sequence of Taqman probe was FAM 5′ TGGATGTGTCTGCGGCGTTTTATCAT 3′ BHQ-1 and the sequence of reverse primer was 5′ GTATCCAGTGCTGCGACCGT 3′.

**Table 1:** Sequences of primers used for evaluation of SHH signaling genes and their regulatory microRNAs.

Accession Number	Gene Name	Primers 5′ → 3′
NM_002046.7	*GAPDH*	Specific forward primer:
		TGGAGTCCACTGGCGTCTTCAC
		RT-PCR primer
		GTCGTATCCAGTGCTGCGACCGTATGGATGTGTCTGCGGCGTTTTATCATGCACTGGATACGACAGGCATTGCTGA
NM_002181.4	*IHH*	Specific forward primer:
		CCCGTCGTGGTGTAGTCATAGAG
		RT-PCR primer
		GTCGTATCCAGTGCTGCGACCGTATGGATGTGTCTGCGGCGTTTTATCATGCACTGGATACGACAGGCTGAGTTGG
NM_001301861.2	*BOC*	Specific forward primer:
		CACTGGCTTGCCTCCTCCTA
		RT-PCR primer
		GTCGTATCCAGTGCTGCGACCGTATGGATGTGTCTGCGGCGTTTTATCATGCACTGGATACGACGCATCATCCGAG
NM_016277.5	*RAB23*	Specific forward primer
		GCTTGTGTGCTCGTGTTCTCTAC
		RT-PCR primer
		GTCGTATCCAGTGCTGCGACCGTATGGATGTGTCTGCGGCGTTTTATCATGCACTGGATACGACACTTCGGCTACTAC
NR_106796.1	*MIR6738*	Specific forward primer
		CGACCTTCTGCCTGCATTCTA
		RT-PCR primer
		GTCGTATCCAGTGCTGCGACCGTATGGATGTGTCTGCGGCGTTTTATCATGCACTGGATACGACCTGGGAG
NR_029712.1	*MIR195*	Specific forward primer
		GCGGCTAGCAGCACAGAAA
		RT-PCR primer
		GTCGTATCCAGTGCTGCGACCGTATGGATGTGTCTGCGGCGTTTTATCATGCACTGGATACGACGCCAATA
NR_030629.1	*MIR509-3*	Specific forward primer
		CGCATACTGCAGACGTGG
		RT-PCR primer
		GTCGTATCCAGTGCTGCGACCGTATGGATGTGTCTGCGGCGTTTTATCATGCACTGGATACGACCATGATTG
NR_004394.1	*RNU6-1*	Specific forward primer
		CTCGCTTCGGCAGCACATATAC
		RT-PCR primer
		GTCGTATCCAGTGCTGCGACCGTATGGATGTGTCTGCGGCGTTTTATCATGCACTGGATACGACGTGTCATCCTTGC

The target specific portion of mRNA and micro-RNA is showed in red bold. Stem-loop sequence is underlined. Tm of the micro-RNA forward primer was increased by adding a tail (green sequences) to the 5′-end of the sequences.

### Statistical analysis

The relative expression of each gene was calculated by StepOne software v2.3 and the 2^−ΔΔCt^ method. Comparisons of results between groups were performed by paired Student’s *t* test. To measure the diagnostic accuracy of Shh signaling genes and regulatory miRNAs in gastric cancer, ROC curve analyses were performed using MedCalc statistical software. A value of *P* < 0.05 was considered as statistically significant.

### Ethics approval

This study was approved by the Ethics Committee of Babol University of Medical Sciences and all procedures were performed in compliance with Helsinki declaration.

## Results

### Clinicopathological features

The clinicopathological features of the gastric cancer patients are summarized in Table 2. Fifty patients with gastric cancer including 38 (76%) male and 12 (24%) female were enrolled in this study. The patients’ mean age and standard deviation at the time of diagnosis was 59.81 ± 14.18 years (34 to 76 years) in females and 67.65 ± 9.5 years (42 to 85 years) in males. There was a significant association between sex and age of gastric cancer patients (*P* = 0.0325). Tumors were mainly located at proximal position of the stomach (cardia, fundus, and body). Overall, 45.5% of tumors were located in the cardia region, and body and antrum regions of the stomach were the second and third most common sites, with 29.5% and 22.7% abundance, respectively. Overall, 74% of tumors were in stages I/II, and 26% in stages III/IV.

**Table 2:** Clinicopathological characteristics of gastric cancer patients.

	Characteristics	%
**Gender**	Male	76
	Female	24
**Age**	< 65 years	42
	≥ 65 years	58
**Differentiation**	Well differentiated	27.1
	Moderately/poorly differentiated	72.9
**Stage**	I	31.9
	II	42.55
	III	23.45
	IV	2.1
**Lymph node metastasis**	Yes	56.8
	No	42.4

### Expression levels of Shh signaling pathway genes and their regulatory miRNAs

**Figure 1:**
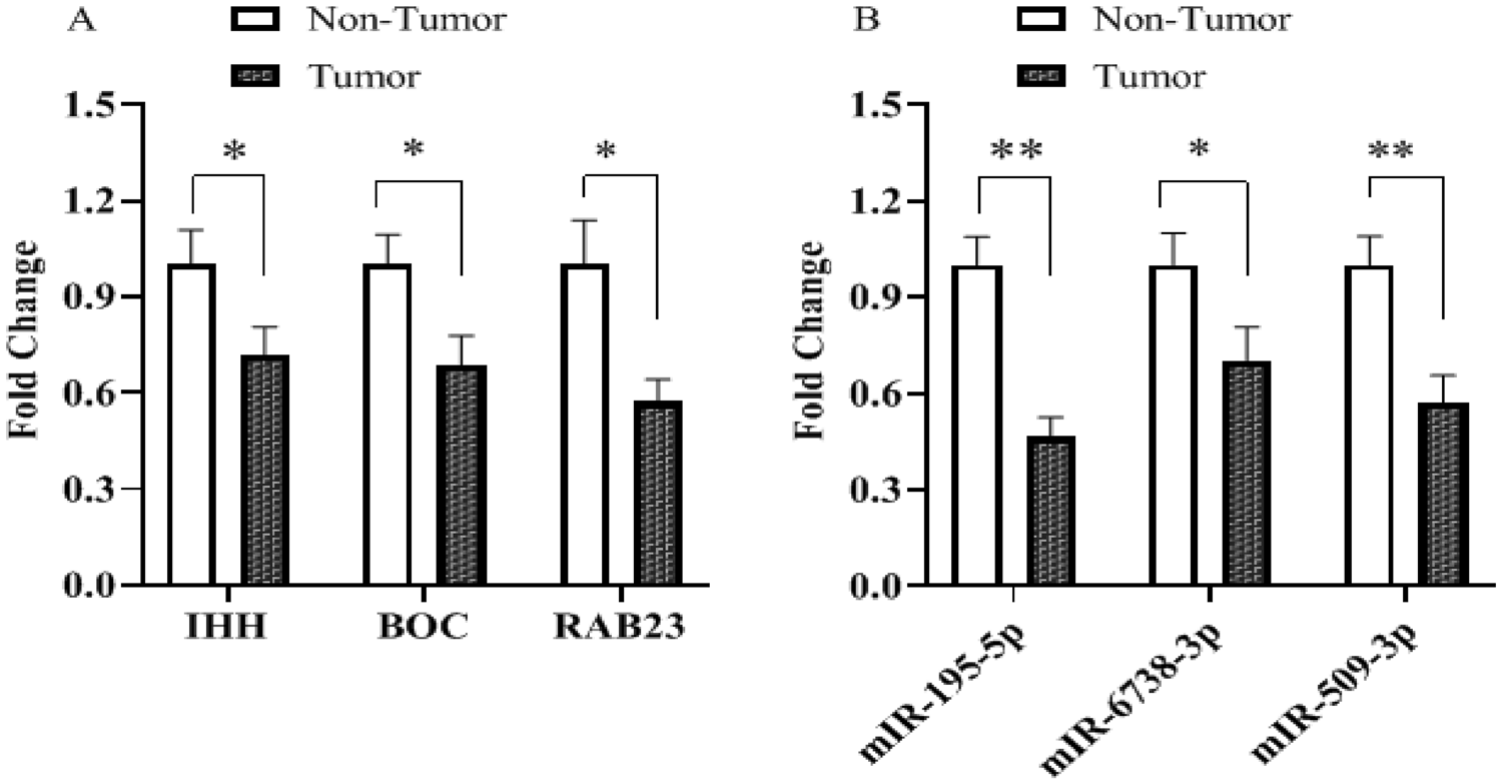
The expression level of Shh genes and their associated miRNAs. (**A**): *IHH*, *BOC*, and *RAB23*; (**B**) *miR-195-5p*, *miR-6738-3p*, and *miR-509-3-5p* in tumoral tissues in comparison with their adjacent non-tumoral counterparts. * and ** indicate significant difference *P* < 0.05 and *P* < 0.01, respectively.

**Figure 2:**
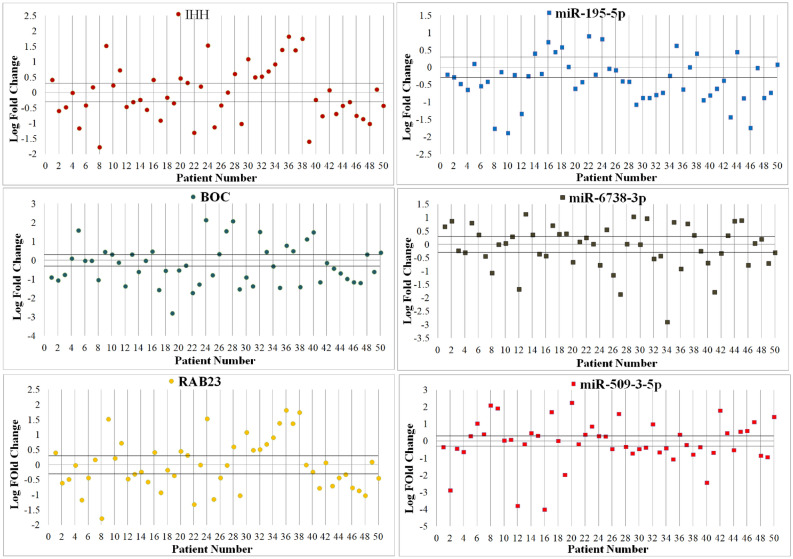
Scatter plot expression of Shh signaling pathway genes and their regulatory miRNAs in tumoral and adjacent non-tumoral tissues. Gene expression variations were considered in 50 gastric adenocarcinoma patients. Each plot represents a person’s gene expression change in tumor tissue in comparison with adjacent normal tissue. Horizontal lines represent cut-off values corresponding to log two-fold changes in expression. The upper and lower parts of the horizontal lines indicate upregulation, and downregulation in the tumoral tissues in comparison with non-tumoral tissues, respectively.

Expression levels of Shh signaling pathway genes (*IHH*, *BOC*, and *RAB23*) and their regulatory miRNAs (*miR-195-5p*, *miR-6738-3p*, and *miR-509-3-5p*) were evaluated in 50 gastric cancer patients using comparative relative real time PCR, and by comparing the expression in tumor tissues with their paired normal counterpart tissues. The results indicated that *IHH, BOC*, and *RAB23* mRNA expression were significantly downregulated (Fig. 1). Similarly, *miR-195-5p, miR-6738-3p*, and *miR-509-3-5p *expression were also decreased significantly in gastric cancer tissues. The mean tumoral tissues expression levels for *IHH*, *BOC*, and *RAB23* were 0.71, 0.68, and 0.57 respectively. Also, the expression levels for *miR-195-5p*, *miR-6738-3p*, and *miR-509-3-5p* in tumoral tissues were 0.46, 0.7, and 0.57 respectively. Scatter plot analysis indicated that *IHH, BOC*, and *RAB23* were significantly down-regulated in 52%, 58%, and 50% of tumoral tissues in comparison with their adjacent non-tumoral counterparts, respectively. Also, out of 50 GC patients, 70%, 54%, and 58% showed statistically significant downregulation of *miR-195-5p*, *miR-6738-3p*, and *miR-509-3-5p* in tumoral tissues in comparison with their adjacent non-tumoral counterparts, respectively (Fig. 2).

### Correlation of Shh signaling pathway genes and their regulatory miRNAs

**Figure 3:**
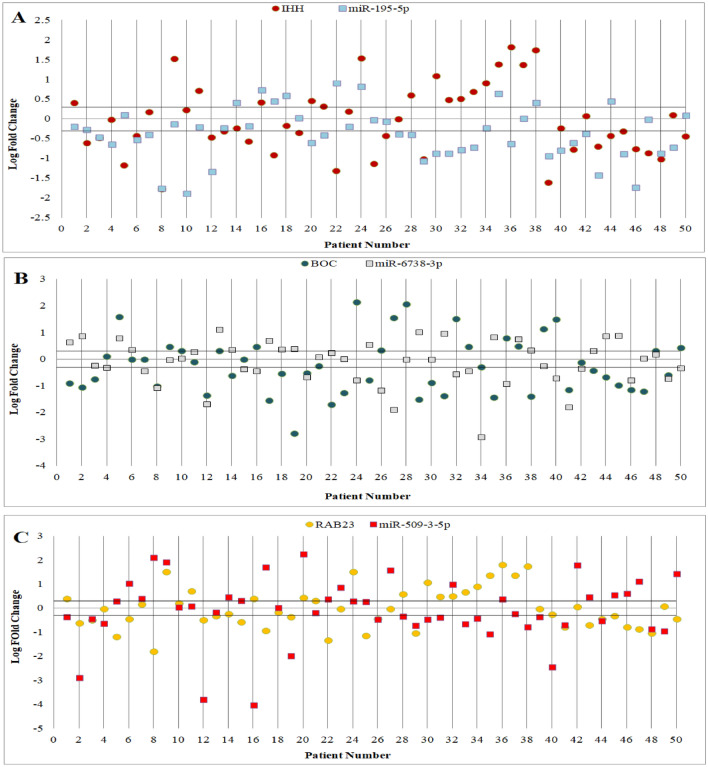
Scatter plot of gene expression in tumoral and adjacent non-tumoral tissues of 50 gastric adenocarcinoma patients. Each plot represents a person’s gene expression change in tumor tissue compared with adjacent normal tissue. Horizontal black lines represent cut‐off values (log2 fold changes in expression). Cases above the upper line demonstrated overexpression in tumoral in comparison with non-tumoral tissues, and those below the lower line demonstrated lower expression levels (differences in expression ≥ 2; *P* < 0.05).

As shown in Fig. 3, analysis of relative expression levels indicated that *IHH*, *BOC*, and *RAB23* were decreased in 48%, 54%, and 42% of gastric cancer tissues while 32%, 30%, and 34% of cases showed *IHH*, *BOC*, and *RAB23* overexpression, respectively. *Mir-195-5p* was downregulated in 52% of cases. Also, 40% and 44% of cases showed *miR-6738-3p* and *miR-509-3-5p* down-regulation, respectively. 23 out of 50 (46%) gastric cancer patients indicated opposite expression pattern for *IHH* and its regulatory miRNA (*miR-195-5p*). Similarly, *BOC* and *RAB23* and their regulatory miRNAs (*miR-6738-3p* and *miR-509-3-5p*) showed opposite expression in 56% and 50% of gastric cancer patients, respectively. Additionally, the miRNA-mRNA interaction between logarithm of fold change (Log FC) of Shh signaling pathway genes and their regulatory miRNA in early stage and advanced stage was calculated using the Pearson correlation test. A weak negative correlation was observed between Shh signaling pathway genes and their regulatory miRNA in different stages.

### Association of clinicopathological features and Shh signaling pathway genes and their regulatory miRNAs

**Figure 4:**
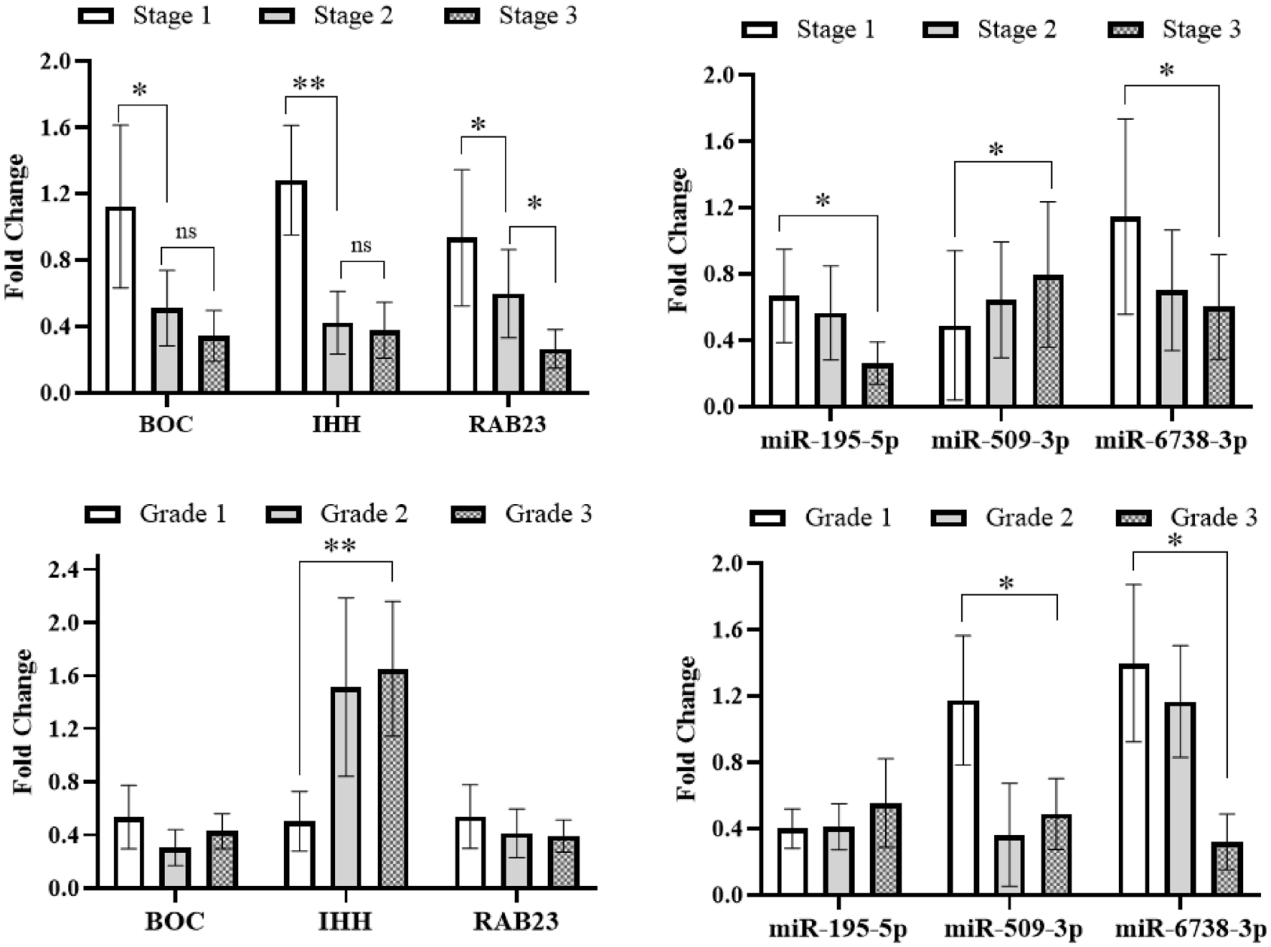
Shh signaling pathway genes and their regulatory miRNAs fold changes in tumor gastric cancer tissues in comparison with their non-tumoral counterparts according to clinicopathological features. * and ** indicates significant difference *P* < 0.05 and *P* < 0.01, respectively.

Association between the expression of Shh signaling pathway genes and their regulatory miRNAs with clinicopathological features in gastric cancer patients is illustrated in Fig. 4. A significantly associated trend was observed between the expression of studied genes and their regulatory miRNAs with TNM stage. The expression of *IHH*, *BOC*, *RAB23*, *miR-195-5p*, and *miR-6738-3p* decreased significantly with more advanced cancer stage, and *miR-509-3-5p* expression was significantly decreased during early stages in gastric cancer patients (*P* < 0.05). Also, *IHH* expression level was associated with histological type, as it was significantly lower in well differentiated gastric adenocarcinomas in comparison with moderately or poorly differentiated adenocarcinoma (*P* < 0.01). Furthermore, *miR-6738-3p* and *miR-509-3-5p* were significantly downregulated in poorly differentiated and moderately/poorly differentiated tumors, respectively (*P* < 0.05) (Fig. 4).

**Figure 5:**
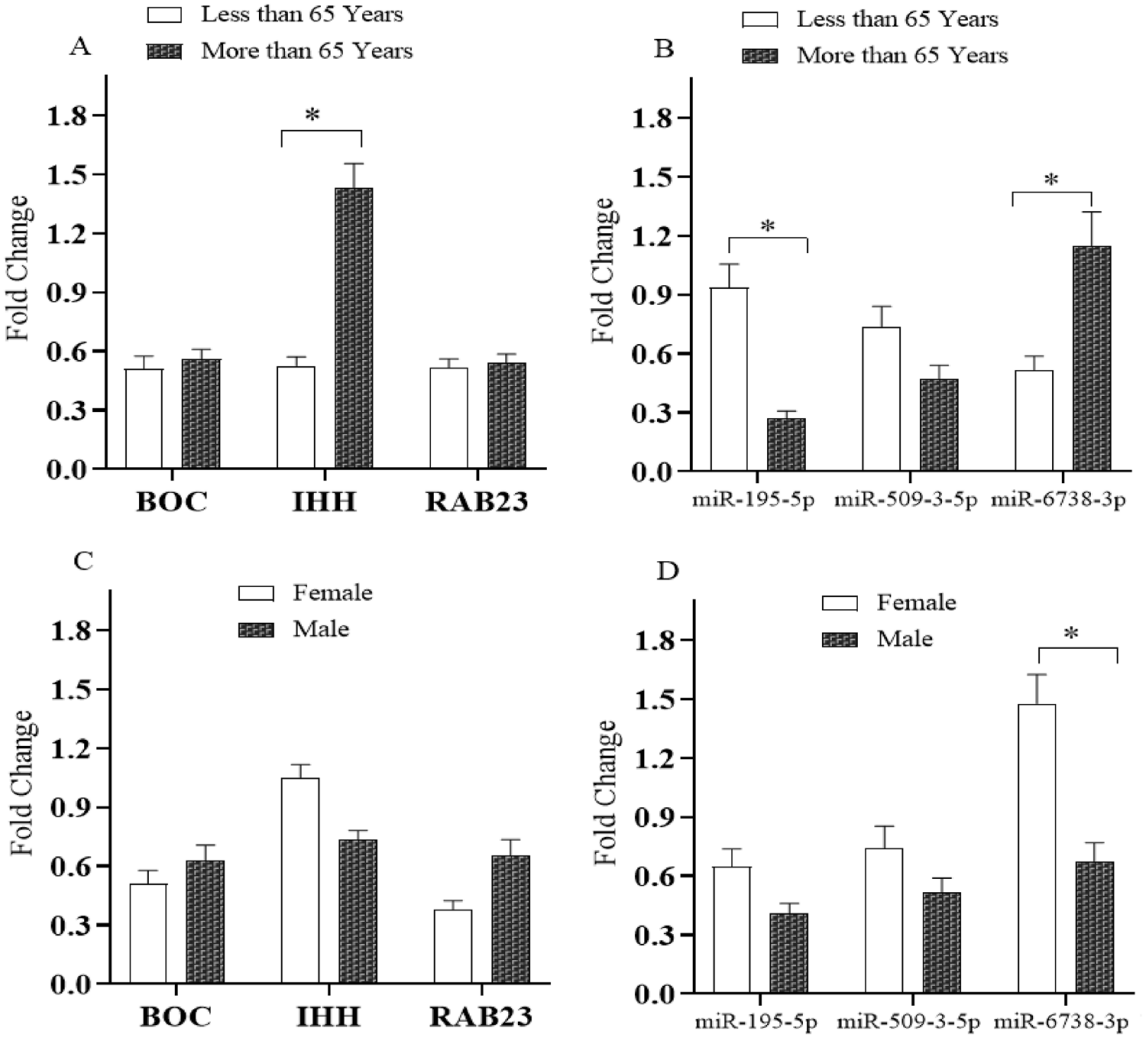
Association of the Shh signaling pathway genes and their regulatory miRNAs expression fold changes in tumoral tissues compared with their adjacent non-tumoral counterparts with age (< 65 years and ≥ 65 years) and gender. * indicates significant difference *P* < 0.05.

Moreover, association study between Shh signaling pathway genes and their regulatory miRNAs according to age groups indicated that *IHH* and *miR-6738-3p* expression was significantly (*P* < 0.05) decreased in gastric cancer patients aged less than 65 years while *miR-195-5p* was significantly *P* < 0.05) down-regulated in gastric cancer patients older than 65 years. Moreover, a statistically significant decreased expression of *miR-6738-3p* was observed in male gastric cancer patients (*P* < 0.05) (Fig. 5).

### Association of the infection status and Shh signaling pathway genes and their regulatory miRNAs expression

**Figure 6:**
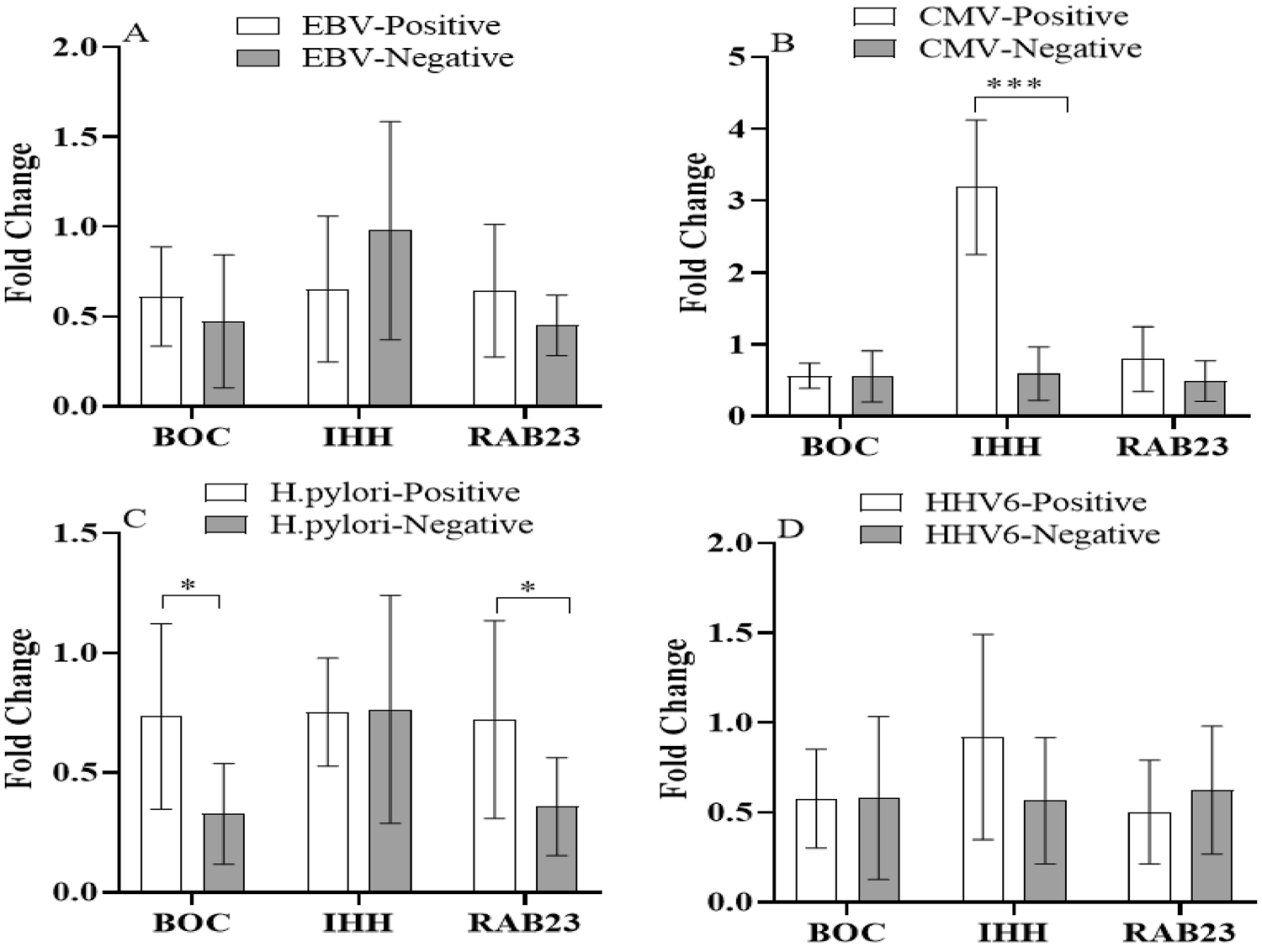
Expression level of Shh signaling pathway genes in non-tumoral and tumoral gastric cancer tissues according to viral infections. * and *** indicate significant difference *P* < 0.05 and *P* < 0.001, respectively. EBV; Epstein-Barr virus, CMV; Cytomegalovirus, HHV6; Human herpesvirus 6.

Furthermore, we investigate the effects of infections on the expression level Shh signaling pathway genes and their regulatory miRNAs in gastric cancer. As shown in Fig. 6, in HCMV positive patients, *IHH* expression was significantly increased (*P* < 0.001). Also, patients with no *H. pylori* infection showed lesser *BOC* and *RAB23* expression in gastric tumoral tissues in comparison with *H. pylori* positive patients (*P* < 0.05). EBV and HHV6 infections had no significant effect on Shh signaling pathway genes and their regulatory miRNAs expression in gastric cancer tissues.

**Figure 7:**
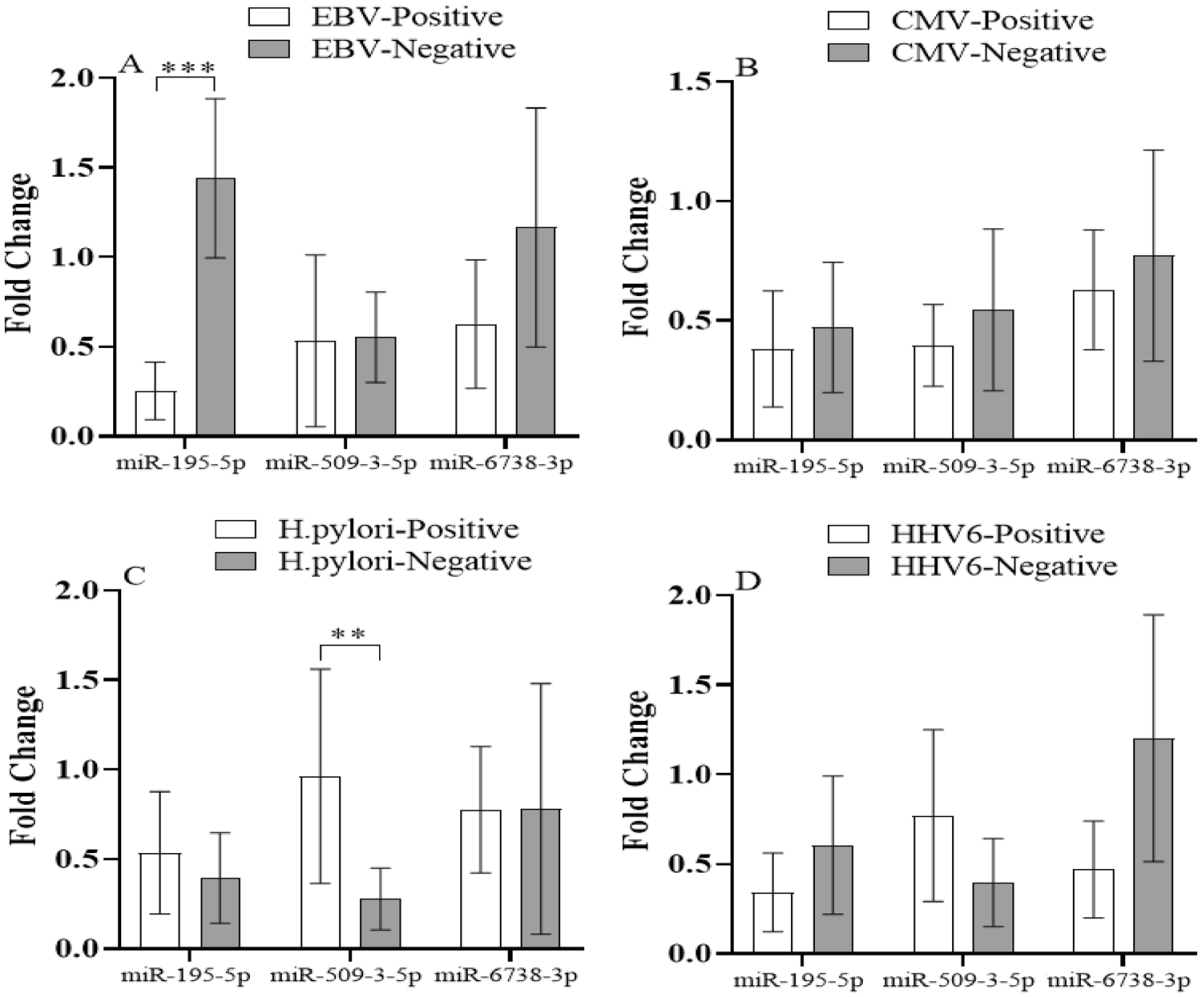
Expression level of Shh signaling pathway regulatory miRNAs in non-tumoral and tumoral gastric cancer tissues according to viral infections. ** and *** indicates significant difference *P* < 0.01 and 0.001, respectively. EBV; Epstein-Barr virus, CMV; Cytomegalovirus, HHV6; Human herpesvirus 6.

Furthermore, *miR-195-5p* expressions were significantly decreased in gastric cancer tissues of EBV positive patients (*P* < 0.001). In addition, there was a significant difference for *miR-509-3-5p* expression level in *H. pylori* positive gastric cancer patients in comparison with *H. pylori* negative patients (*P* < 0.01). HCMV infections had no significant effect on Shh signaling pathway regulatory miRNAs expression (Fig. 7).

### Receiver operating characteristic (ROC) curve analysis

**Figure 8:**
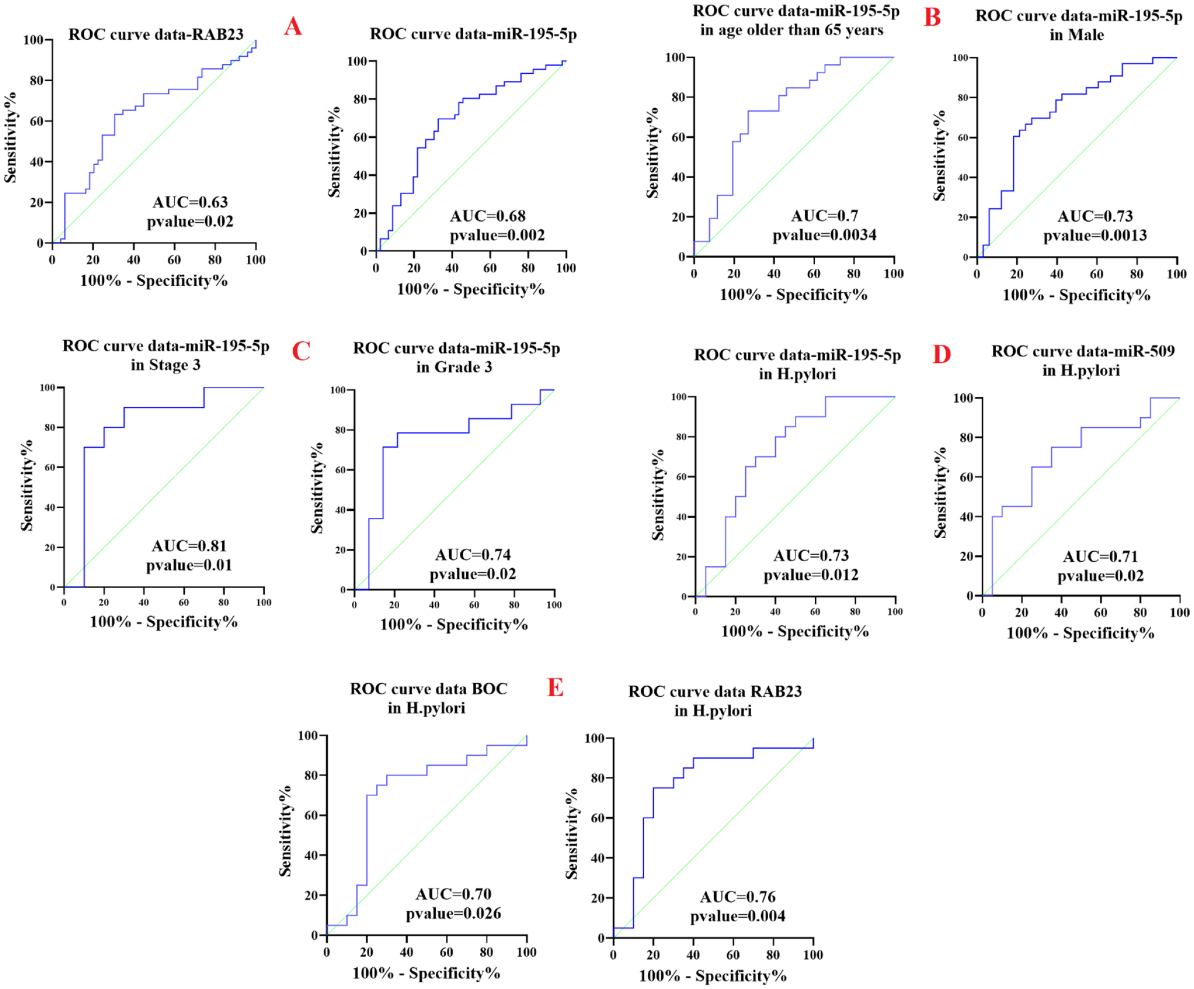
Receiver operating characteristics (ROC) curves for gene expression of potential biomarkers in gastric cancer. (**A**): The AUC of *RAB-23* and *miR-195-5p* in gastric canccer was 0.63 and 0.68, respectively (**B**): The AUC of *miR-195-5p* in gastric cancer patients older than 65 years and male patients were 0.7 and 0.73, respectively (**C**): The AUC of *miR-195-5p* in advanced stage and grade in gastric cancer patients were 0.81 and 0.74, respectively (**D**): The AUC of *miR-195-5p* and *miR-509* in *H.pylori* negative gastric cancer patients was 0.73 and 0.71, respectively (**E**): The AUC of *BOC* and *RAB23* in *H.pylori* negative gastric cancer patients was 0.7 and 0.76, respectively. AUC: the area under the curve.

ROC curve analysis used to reveal whether studied genes and their regulatory miRNAs can serve as diagnostic biomarkers (Fig. 8)^35^. As it is evident, total area under the curves (AUCs) of *RAB23* (AUC = 0.63, sensitivity 71% and specificity 51%, *P* = 0.02) and *miR-195-5p* (AUC = 0.68, sensitivity 80% and specificity 54%, *P* = 0.002) were > 60%, suggesting that *RAB23* and *miR-195-5p* can serve as diagnostic biomarkers for distinguishing patients with gastric cancer from healthy controls. ROC curve analysis was used to evaluate the predictive potential of Shh signaling pathway genes and their regulatory miRNAs with clinical parameters including gender, age, tumor grade, and TNM stage. The ROC analyses indicated that *miR-195-5p* expression levels were able to reliably distinguish late stages and grade from early stages and grade, with AUC 0.81 (sensitivity 90% and specificity 70%, *P* = 0.01) and 0.74 (sensitivity 78.57% and specificity 71.43%, *P* = 0.02), respectively. In addition, differences in the *miR-195-5p* expression level could successfully discriminate gastric cancer males from females (AUC = 0.73, sensitivity 81.8% and specificity 57.5%, *P* = 0.0013), as well as those belonging to the age group older than 65 years from younger than than 65 years (AUC = 0.73, sensitivity 80.77% and specificity 57.69%, *P* = 0.003). Furthermore, ROC curve analysis was applied to study the potential of Shh signaling pathway genes and their regulatory miRNAs as a biomarker in gastric cancer patients infected with EBV, CMV, HHV6, and *H. pylori*. ROC curve analysis showed good predictive accuracy for *BOC, RAB23a, miR-195-5p*, and *miR-509-3-5p* in discriminating *H. pylori* infected patients from uninfected patients (AUC of 0.703 for *BOC,* sensitivity 80% and specificity 70%, *P* = 0.02; AUC of 0.76 for *RAB23a*, sensitivity 90% and specificity 65%, *P* = 0.0045; AUC of 0.73 for *miR-195-5p*, sensitivity 80% and specificity 60%, , *P* = 0.012; AUC of 0.71 for *miR-509-3-5p*, sensitivity 75% and specificity 65%, *P* = 0.02).

### Discussion

Gastric cancer is among the five most frequently diagnosed cancers, and is highly heterogeneous. Accumulating evidence strongly indicate that aberrant activation of multiple signaling pathways can contribute to gastric cancer development. Consequently, cancer stem cells (CSCs) are key driving cells for growth and metastasis of this tumor type. It has been demonstrated that Shh signaling pathway is implicated in maintaining the pluripotency of CSCs, and aberrant activation of this pathway is associated with the development and progression of various types of cancer. In this study, we investigated the clinicopathological features of gastric carcinoma as well as expression levels of Shh signaling pathway genes and their regulatory miRNAs in gastric cancer patients. Although gastric cancer is common in both sexes, its incidence is higher in males, and is more frequently observed in younger female patients^36–38^. Our demographic findings are consistent with these reports. We also investigated the expression level of Shh signaling genes including *IHH*, *BOC*, and *RAB23* in gastric cancer patients. Remarkably, we observed that *IHH* expression was decreased in tumoral tissues in comparison with adjacent non-tumoral tissues. Also, *IHH* expression strongly correlated with the stage and grade of malignancy as well as with CMV infection. *IHH* is one of the three protein ligands in the mammalian hedgehog signaling pathway, and plays an essential role in bone growth and differentiation. The expression level of *IHH* was found to be upregulated in certain tumors such as basal cell carcinoma, pancreatic cancer, and medulloblastomas^39^. Also, immunohistochemical study indicated that IHH expression was increased in pancreatic ductal adenocarcinoma in comparison with paracancer tissue and benign lesions, and this expression was associated with tumor grade, lymph node metastasis, tumor invasion, and poor overall survival^40^. In contrast, loss of IHH expression can promote the development of dysplasia in colon carcinogenesis via Wnt signaling pathway^41^. Also, epidermal deletion of IHH can promote squamous skin tumor formation and increased malignant tumor progression and metastasis as well as prolonged loss of IHH expression leads to inflammation and mucosal damage^42^. In addition, stromal activation of Hh signaling pathways by IHH suppresses tumor growth and metastases through angiogenesis and reduction of reactive oxygen species (ROS) activity. However, the tumor suppressor or oncogenic role of IHH in cancer is controversial. Relatively, our data demonstrated a decrease in *BOC* mRNA levels in tumoral tissues, and this expression was associated with the tumor’s stage and *H.pylori* infection. BOC is a co-receptor in Shh signaling pathway and a component of cell-surface receptor complex that mediates cell–cell interactions. However, its relative contribution to cancer risk is currently unknown. The hedgehog co-receptors including GAS1, CDON, and BOC modulate the levels of HH responsiveness in pancreatic fibroblasts, and loss of BOC and GAS1 was shown to reduce HH activity while promoting pancreatic tumor growth through the induction of angiogenic factors^43^. In addition, BOC may induce DNA damage and promote progression of early medulloblastoma to advanced tumors via increasing the incidence of loss of heterozygosity (LOH) of its coreceptor PTCH1^44^. Moreover, our results indicated that *RAB23* was downregulated in 42% of gastric cancer tissues while it was overexpressed in 34%of cases. Also, the expression of *RAB23* was significantly associated with more advanced cancer stage and *H. pylori* infection. RAB23 is recognized as a negative regulator of the Shh signaling pathway, and also as the target of many proteins involved in cancer development. However, it remains controversial whether RAB23 acts as an oncogene or tumor suppressor. Although more and more studies have introduced RAB23 as an oncogene in variety of human cancers, there is some evidence indicating that RAB23 plays a tumor suppressive role during carcinogenesis. RAB23 through interaction with SUFU can inhibit *GLI* transcriptional activities and its nuclear localization. In addition, overexpression of *RAB23* inhibits breast cancer cells viability and proliferation, and induces cell apoptosis^45^. Moreover, transient overexpression of miR-367 in medulloblastoma cells caused decreased *RAB23* expression resulting in increased medulloblastoma cell proliferation^46^.

It is now established that both genetic and epigenetic alterations contribute to gastric cancer development. Changes in miRNAs expression, as epigenetic modulators, via regulation of cancer-related genes have a primary role in cancer onset and progression. We studied the Shh signaling pathway regulatory miRNAs by in silico analysis, and experimentally validated the expression levels of these miRNAs in gastric cancer patients. Through in silico analysis, we identified three miRNAs, including miR-195-5p, miR-6738-3p, and miR-*509-*3-5p, which could bind to the 3′ UTRs of Shh signaling genes and modulate the hedgehog pathway. While there is no evidence supporting a role for miR-6738-3p in cancer development, our findings showed that miR-6738-3p expression was decreased in tumoral tissues in comparison with adjacent non-tumoral tissues. Also, its expression was associated with the clinicopathological features of gastric cancer patients including stage, grade, gender, and age. Our in silico analysis predicted that mir-195-5p binding site was located in the 3′ UTR of *IHH*, and experimental data indicated that mir-195-5p was significantly down-regulated in tumor samples in comparison with their adjacent non-tumoral tissues. Also, mir-195-5p expression was strongly associated with the advanced cancer stage and age of gastric cancer patients, and correlated with EBV infection. Mir-195-5p is one of the well-studied miRNA that is strongly connected with various types of cancer including those of digestive system, respiratory system, urinary system, reproductive system, bone, brain, head and neck, skin, and endocrine cancer. Nevertheless, despite its strong tumor suppressive effects, there is evidence that miR-195 has an oncogenic role in some cancers. Therefore, whether mir-195-5p functions as a tumor suppressor or oncogene is still under debate. It has been reported that miR-195-5p had significant effect on oncogenicity in various types of cancer through binding to complementary sequences in crucial genes of signaling pathways. In stomach cancer, evidence had indicated that mir-195-5p is able to sensitize chemotherapeutic resistant gastric cancer cells to 5-fluorouracil and cisplatin by directly targeting *ZNF139* and AKT3, respectively^47,48^. Also, Epstein–Barr virus-encoded miRNA *BART1* through regulating the abundance of cellular tumor suppressive miRNAs such as miR-150, miR-152, and miR-195 was shown to induce tumor metastasis in nasopharyngeal carcinoma. This result further strengthens our observation that miR-195 expression was significantly decreased in gastric cancer tissues of EBV positive patients^49^.

Mir-509 is one of the anti-oncogene miRNAs that was reported to be downregulated in many prevalent human cancers. Mir-509 functions as a tumor suppressor in pancreatic and breast cancer via targeting MDM2 proto-oncogene (*MDM2)* and superoxide dismutase 2 (*SOD2)*^50,51^. Also, miR-509 as epithelial-mesenchymal transition miRNA was shown to induce the expression of E-cadherin, and inhibit cell motility and invasion^52^. In addition, miR-509 can inhibit cell motility and invasion through targeting tribbles pseudokinase 2 (*TRIB2)* in osteosarcoma^53^. Our findings showed that miR-509 was significantly down-regulated in tumor tissues in comparison with their adjacent non-tumoral tissues, and lower miR-509 expression in tumoral tissues was associated with high tumor grade, stage, and *H. pylori* infection. In line with our results, a similar study demonstrated that lower miR-509*-*3-5P expression was associated with advanced tumor stage and poor differentiation in gastric cancer tissues. Additionally, this lower expression promoted the migration and invasion abilities of gastric cancer cells by targeting podocalyxin like (*PODXL)* gene^54^. However, our finding is in disagreement with another study indicated miR-509 upregulation in *H. pylori*-negative gastric cancer^55^.

Gastric cancer can be considered a complex disease that is influenced by multiple genes, miRNAs, and environmental factors. Finding valuable biomarkers for the diagnosis and prognosis of gastric cancer is difficult. Therefore, using deep convolutional neural network learning method is a very promising way to predict disease risk based on biomarkers^56^. Our ROC curve analysis indicated that *mir-195* can be considered a good biomarker as it was significantly related to several clinicopathological features of gastric cancer including stage, grade, age, gender, and infection status. In conclusion, the present study demonstrated that the expression level of Shh signaling pathway genes and their regulatory miRNAs were significantly associated with gastric cancer. Also, the expression level of these genes was significantly associated with clinicopathological features including tumor stage, grade, age, gender, and infection status in gastric cancer patients. Although Hh signaling inhibitors have been developed, and two inhibitors, vismodegib and sonidegib, have been approved by the U.S. Food and Drug Administration (FDA) to treat basal cell carcinoma and medulloblastoma, none of them have been approved for gastric cancer. Thus, more researches to elucidate factors regulating Shh signaling pathway as well as their detailed mechanism of action in gastric cancer are necessary. Also, it is well known that immune regulations may affect gastric cancer stage development, and patients with common variable immunodeficiency (CVID) have a high risk of gastric cancer^57^. Recently, in vivo study indicated that immunodeficiency can promote adaptive alterations of host gut or tissue-based microbiome^58^. Therefore, additional studies on alterations of gastric mucosal immunity and microbiota and effects on signaling pathways are needed to better understand the gastric carcinogenesis.
