# Author Correction: Association of sonic hedgehog signaling pathway genes IHH, BOC, RAB23a and MIR195-5p, MIR509-3-5p, MIR6738-3p with gastric cancer stage

**DOI:** 10.1038/s41598-021-95379-8

**Published:** 2021-08-02

**Authors:** Sadegh Fattahi, Novin Nikbakhsh, Mohammad Ranaei, Davood Sabour, Haleh Akhavan-Niaki

**Affiliations:** 1 Cellular and Molecular Biology Research Center, Health Research Institute, Babol University of Medical Sciences, Babol, Iran; 2 Department of Surgery, Rouhani Hospital Babol University of Medical Sciences, Babol, Iran; 3 Department of Pathology, Rouhani Hospital, Babol University of Medical Sciences, Babol, Iran; 4 Department of Genetics, Faculty of Medicine, Babol University of Medical Sciences, Babol, Iran

Correction to: *Scientific Reports* 10.1038/s41598-021-86946-0, published online 02 April 2021

In the original version of this Article Haleh Akhavan-Niaki was incorrectly affiliated with ‘Department of Surgery, Rouhani Hospital Babol University of Medical Sciences, Babol, Iran’. The correct affiliations are listed below.

Cellular and Molecular Biology Research Center, Health Research Institute, Babol University of Medical Sciences, Babol, Iran.

Department of Genetics, Faculty of Medicine, Babol University of Medical Sciences, Babol, Iran.

The original Article has been corrected.

